# Influence of Dietary Protein Content on the Nutritional Composition of Mealworm Larvae (*Tenebrio molitor* L.)

**DOI:** 10.3390/insects14030261

**Published:** 2023-03-06

**Authors:** Nina Kröncke, Rainer Benning

**Affiliations:** Institute of Food Technology and Bioprocess Engineering, University of Applied Sciences Bremerhaven, An der Karlstadt 8, 27568 Bremerhaven, Germany

**Keywords:** *Tenebrio molitor*, nutritional composition, insect diet, amino acids, insect rearing

## Abstract

**Simple Summary:**

Protein-rich insects are becoming more popular as livestock feed alternatives to fish and soy meal. A variation of different diets was used to rear mealworm larvae for the purpose of influencing their chemical composition. How dietary protein content affects larval protein and amino acid composition and growth rate was primarily investigated. Experimental diets used wheat bran as the control substrate, while different types of flour, notably pea protein, rice protein, sweet lupine, and cassava, along with potato flakes, were mixed with the wheat bran. Each substrate and larva were then analyzed for moisture, protein, and fat content, as well as the amino acid profile. A supplementation of pea and rice protein was determined to be most beneficial in terms of high protein yield and lower fat content in larvae. Cassava flour and wheat bran mixed together produced the highest amount of amino acid and essential amino acid content. Additionally, dietary fats and carbohydrates were found to have a greater influence on larval composition than protein content. This research could improve future formulations of artificial diets for *Tenebrio molitor* larvae.

**Abstract:**

The use of insects as livestock feed is becoming increasingly accepted because they provide an important source of protein. The purpose of this study was to investigate the chemical composition of mealworm larvae (*Tenebrio molitor* L.) reared on a range of diets that differed in nutritional composition. Focus was placed on the influence of dietary protein content on larval protein and amino acid composition. For the experimental diets, wheat bran was chosen as the control substrate. The following types of flour-pea protein, rice protein, sweet lupine, and cassava, as well as potato flakes, were mixed with wheat bran and used as the experimental diets. An analysis of the moisture, protein, and fat content was then carried out for all diets and larvae. Furthermore, the amino acid profile was determined. It was shown that supplementing the feed with pea and rice protein was most suitable in terms of high protein yield in larvae (70.9–74.1% dry weight) with low fat content (20.3–22.8% dry weight). The total amino acid content was highest in larvae that were fed with a mixture of cassava flour and wheat bran (51.7 ± 0.5% dry weight), as well as the highest content of essential amino acids (30.4 ± 0.2% dry weight). Moreover, a weak correlation between larval protein content and diet was identified, yet a stronger influence of dietary fats and carbohydrates on larval composition was found. This research could result in improved formulations of artificial diets for *Tenebrio molitor* larvae in the future.

## 1. Introduction

In recent years, insects have been considered a viable alternative and sustainable source of nutrients for animal feed and human consumption, and rearing insects has drawn considerable scientific attention [1,2,3]. There are a number of insect species that have the potential to be used in industrial settings and to be produced on a large scale for commercial purposes, including the yellow mealworm (*Tenebrio molitor* L., Coleoptera: Tenebrionidae). Mealworm larvae are often fed to pets (e.g. reptiles) [4] because of their nutritional value, whereas they have also been tested for use in pig [5,6] and poultry [7,8] diets, as well as in artificial diets for mass-rearing beneficial organisms, including a predatory lady beetle (*Coleomegilla maculata* De Geer, Coleoptera: Coccinellidae) [9]. However, insects are also suitable for human nutrition. Worldwide, approximately 2 billion people consume insects as part of their traditional diet [10], and there are a number of products, such as cereal bars, pasta, chocolate, etc., on the market which contain insects [10]. Insects are rich in proteins and their nutritional composition can be strongly influenced through adapted feeding [11,12]. A variety of factors affect the nutritional value, as well as the growth, of insect species, including their diet, sex, developmental stage, species, growth environment, and measurement methods [13]. The growth and the performance of insects are heavily influenced by their diet. Consequently, the development of effective artificial diets has been considered one of the most important components of insect-producing systems, such as producing phytophagous insects [14,15], beneficial arthropods [16], or insects for food and feed [17,18]. Considerable efforts have been geared towards developing artificial diets for *Tenebrio molitor* that maximize biomass production of larvae while simultaneously reducing their development time. For example, a variety of food by-products was fed to mealworm larvae, and their survival rate, feed conversion efficiency, and development time varied significantly [11]. Mealworm larvae have a relatively high protein content (50–60% on a dry matter basis) [19,20] and high lipids content (20–34% on a dry matter basis) [20,21,22], and are a reliable source of essential amino acids [23]. A major challenge for the insect-producing industry is to achieve cost-effective, efficient, and sustainable insect production for food and feed through the development of feeds that support and maximize insect development and growth [24]. A species-specific diet should be designed for each insect species to meet their nutritional requirements. Diets should also be designed for each life stage for maximizing the total larval biomass production and enhancing adult reproduction performance [10]. Research studies of the nutritional requirements of *Tenebrio molitor* date back to the 1950s when diets containing 80–85% carbohydrates were proposed as a suitable diet for its larvae [25]. However, detailed work needed to be conducted to further understand the nutritional requirements of Tenebrio molitor larvae in the following decades [26,27,28,29]. It is possible to feed *Tenebrio molitor* larvae only with bran, which contains all the required nutrients, but not in the ideal proportions, thus making it necessary to supplement the diet [30]. The most common substrate composition in mealworm rearing laboratory facilities and in the industry consists of bran along with a water source (e.g., apples, carrots, or cabbage), and/or a source of protein (e.g., soy protein, beer yeast, or casein) [31]. As a result, many researchers suggest that diet may be used to manipulate the body composition of insects—and hence the nutritional quality of their bodies—to meet various nutritional requirements [11,32,33]. However, the diets are not nutritionally formulated specifically for this insect species, as is the case for other livestock (e.g., pig or poultry) [24]. In spite of this, compound animal feeds have historically been used to produce *Tenebrio molitor*, originally designed for other traditional farm animals [24]. Therefore, several previous research studies evaluated different diets that can be used for producing *Tenebrio molitor* larvae in mass quantities. For instance, a diet containing peanut oil, canola oil, salmon oil, dry potato flour, soy protein, and dry egg white was evaluated as a nutritional supplement for *Tenebrio molitor* [34]. Diets with higher protein and lipid contents significantly improved most biological parameters determined, in comparison to diets containing a high carbohydrate content [34]. The development of nutritionally balanced diet mixtures for the mass production of mealworm larvae could be improved by analyzing the growth and performance of this species on single component and compound feeds. In contrast to oligophagous storage pests, which prefer eating only a few specific foods, *Tenebrio molitor* has a huge range of food preferences. The effects of supplementation and differences in dietary nutritional composition on *Tenebrio molitor* growth, feed conversion, development, survival, and nutritional composition have been reported in previous research studies [11,32,35,36]. However, it is still not entirely clear what protein content and amino acid profile is optimal for rearing *Tenebrio molitor* larvae and which protein source is most suitable for insect growth. In addition, the influence of the dietary amino acid content on larval amino acid content was determined. This could lead to a better understanding of the importance of dietary protein content on the growth and nutritional composition of *Tenebrio molitor* larvae used for mass rearing. In this research study, the objective of the present work was to obtain basic information on the suitability of feeding substrates for larval development, and to analyze the effect of substrates with varying protein content on the protein, lipid, and amino acid content of *Tenebrio molitor* larvae.

## 2. Materials and Methods

### 2.1. Insect Samples

The experiment was carried out at the University of Applied Sciences Bremerhaven with in-house bred *Tenebrio molitor* larvae. A constant climate chamber (HPP 110, Memmert, Schwabach, Germany) was used to raise eight-week old mealworm larvae growing at 27 °C and 75% relative humidity. Larvae were fed wheat bran ad libitum until they were selected for the experiment. Each experimental group contained 100 larvae with an average starting weight of 6.4 ± 0.0 mg per larva with an age of eight weeks, and these were then put into 400 mL beakers and weighed (ADB 200-4, Kern & Sohn GmbH, Balingen-Frommern, Germany) at the start of the experiment and at the end to measure biomass growth. Mealworm larvae were separated using a sieve after a five-week experiment from the frass and feed leftovers to determine their food conversion efficiency. The survival rate was determined by counting all dead larvae at the end of the investigation. After 24 h of starvation, larvae were frozen for 48 h at −21 °C in a commercial freezer (HAS 47520, Beko, Neu-Isenburg, Germany) and stored until their moisture, protein, fat and amino acid contents were analyzed.

### 2.2. Feeding Groups

A variety of substrates, with different protein sources, were selected because of the variations in their nutrient compositions (see Table S1 in Supplementary Materials). Treatments were combined based on their macronutrient content, as well as on the differences in amino acid content. A substrate that contains a high and low proportion of protein should be used for the diet. This is in order to examine the influence the substrate has on the growth and nutritional composition, specifically on the protein and amino acid content, of *T. molitor* larvae. According to the nutritional composition of the experimental diets, the following ingredients were selected (Table 1): pea protein flour (Raab Vitalfood GmbH, Rohrbach, Germany), rice protein flour (Raab Vitalfood GmbH, Rohrbach, Germany), sweet lupine flour (Natura-Werk Gebr. Hiller GmbH & Co. KG, Hannover, Germany), cassava flour (EWL Naturprodukte Handelsagentur AG, Randbach-Baumbach, Germany), potato flakes (Mühlenlädle, Kirchberg an der Murr, Germany), and wheat bran (Roland Mills United GmbH & Co. KG, Bremen, Germany). This study focused primarily on the protein and amino acid content of the diets, so groups with different protein contents were created by mixing the substrates with wheat bran. The treatments were named according to the used substrates and their protein content (e.g., PPF80: group consists of pure pea protein flour with a protein content of 80%). Pure wheat bran (WB) was used as the control group. For each feeding group, five replicated beakers of 10 g of diet (2.9 mg dry matter per day and per larvae) were provided, along with 3 g of carrot per week as a water source.

The nutrient composition (Table 2) and the amino acid contents (Table 3) of the diets used for the feeding experiment are presented below. The amino acid content of the single substrate can be viewed in Supplementary Materials (Table S2). The main focus of this study was on the variation of the dietary protein content, meaning the substrates were mixed so the protein content varied between 10 and 80%.

### 2.3. Growth Parameters

Growth parameters and food utilization were calculated at the end of the feeding trial according to Waldbauer (1968) [37]. Larval weight gain per larva (LWGpL in mg) is an increase in live weight gain per unit of time and was determined by subtracting the initial larval weight from the accumulated weight of live larvae, divided by the number of larvae at the end of the experiment. Food conversion ratio (FCR) was calculated on a fresh basis by dividing the amount of feed consumed by the weight gained. Specific growth rate (SGR in % per day) is a coefficient that measures the percentage increase in larvae weight per day and was calculated by subtracting the logarithmic initial body weight from the logarithmic final body weight, divided by the number of experimental days. The protein efficiency ratio (PER) was evaluated to determine the quality of the diets and the efficiency of the larvae converting the protein from the diets into body weight. PER was calculated by dividing the weight gain of the larvae by the total protein fed on a fresh matter basis.

### 2.4. Analysis of Nutritional Composition

*Tenebrio molitor* larvae and substrates were analyzed for nutritional composition as previously described [38]. The moisture content of the samples were determined according to DIN EN 25663 and the Association of German Agricultural Analytic and Research Institutes [39] by drying them at 103 °C for 4 h in a drying oven (U10, Memmert, Schwabach, Germany). The Kjeldahl method was used to measure the protein content of the samples, which was calculated according to DIN EN 25663 and the Association of German Agricultural Analytic and Research Institutes by multiplying the nitrogen content with a factor of 6.25 [39]. The Soxhlet method, as described by the Association of German Agricultural Analytic and Research Institutes [39], was used to determine the fat content of the samples using petroleum benzine as an extraction solvent. Fiber content was analyzed as described by the Association of German Agricultural Analytic and Research Institutes [39]. The samples were incinerated in a muffle furnace (M 110, Brabender, Germany) at a temperature of 550 °C for 30 min. The ash content of the samples was measured according to the Association of German Agricultural Analytic and Research Institutes [39] using a muffle furnace (M 110, Brabender, Germany) for 4 h at 550 °C. The content of carbohydrates was calculated from the difference to 100% of all other constituents. The content of nutrients was calculated as percentages based on fresh weight.

### 2.5. Analysis of Amino Acid Content

The amino acid content of feeding substrates and mealworm larvae were analyzed using high-performance liquid chromatography (HPLC) combined with o-phthalaldehyde (OPA) derivatization as described by Roth (1971) with some modifications [40]. Feeding substrates were homogenized using a Moulinette DPA141 (Groupe Seb Deutschland GmbH, Frankfurt am Main, Germany), and larvae were freeze-dried (Alpha 1–2 LDplus, Martin Christ Gefriertrocknungsanlagen GmbH, Osterode am Harz, Germany) for 24 h before analysis. 

For hydrolysis, about 10–15 mg of the sample was placed in 20 mL glass tubes, then 2 mL H_2_O (ultrapure, Purelab Flex 2, Elga LabWater, Celle, Germany) and 2 mL hydrochloric acid (37%, fuming, Carl Roth GmbH & Co. KG., Karlsruhe, Germany) were added, and then hydrolyzed in a heating oven (Heraeus, Hanau, Germany) at 110 °C for 20 h. After hydrolysis, samples were transferred to a 25 mL measuring cylinder, adjusted to pH 8.5 by adding a boric acid-sodium hydroxide solution (pH 14, Carl Roth GmbH, Karlsruhe, Germany, ice cooling) and diluted to 25 mL with H_2_O (ultrapure). The hydrolysate was then pipetted into 1 mL vials and centrifuged at 15,000 rpm for 5 min (Universal 320 R, Hettich, Tuttlingen, Germany). The supernatant was diluted 1:40 with H_2_O (ultrapure), filtered (0.20 µm; Rotilabo, Carl Roth GmbH, Karlsruhe, Germany), and stored at 4 °C until measurement was carried out.

For derivatization, 14 µL of OPA reagent (phthaladehyde Reagent, Sigma-Aldrich Chemie GmbH, Taufkirchen, Germany) was mixed with 50 µL of sample and incubated for 3 min at room temperature in a HPLC vial.

The chromatographic system (Shimadzu LC 10, Shimadzu Deutschland GmbH, Duisburg, Germany) included a chromatographic column (Kinetex C18 HPLC column, 2.6 µm, 150 × 4.6 mm with guard, Phenomenex Ltd., Aschaffenburg, Germany) and a fluorescence detector (RF-10A, Shimadzu Deutschland GmbH, Duisburg, Germany). The samples were separated at 32 °C using a solvent gradient. Eluent A was a 20 mM sodium acetate buffer (1.64 g/L) adjusted with acetic acid (10%, Carl Roth GmbH & Co. KG, Karlsruhe, Germany) to pH 6.0. Eluent B was a 100 mM sodium acetate buffer (1.64 g/L) adjusted with 21 mL acetic acid (10%, Carl Roth GmbH & Co. KG, Karlsruhe, Germany), 400 mL acetonitrile (40%, Rotisolv, Carl Roth GmbH & Co. KG., Karlsruhe, Germany), and 400 mL methanol (40%, Carl Roth GmbH & Co. KG, Karlsruhe, Germany) to pH 6.0. The flow gradient conditions are presented in Table 4.

For detection, detector wavelengths were set at 330 nm for excitation and 445 nm for emission. The mixture was compounded during two cycles and finally injected with an injection volume of 3–10 µL. Amino acid quantification was performed by the internal amino acid standard solution (AAS18) from Sigma-Aldrich Chemie GmbH. 

### 2.6. Statistics

Statistical tests for normality (Shapiro–Wilk test) and homogeneity of variance (Bartlett’s test) were performed with five independent replications. A one-way ANOVA and a Tukey–Kramer post hoc test were performed using SigmaPlot 12.5 (Systat Software Inc., Düsseldorf, Germany) to determine the statistical significance of the results. A 95% confidence interval was used to declare significant differences (*p* < 0.05). An orthogonal contrast analysis was performed to test the linear and quadratic effects among the means of the growth performance, feed and protein efficiency ratio, survival rate, and nutritional composition of *Tenebrio molitor* larvae. Moreover, linear regression analysis was performed to measure the correlation between the protein content and the individual larval weight. A similar process was followed to examine whether larval protein and fat content correlated with the nutrient content of the diets.

## 3. Results

### 3.1. Growth Parameters

In Table 5, larval growth is represented as larval weight gain per larva (LWGpL) and specific growth rate (SGR), as well as the feed conversion rate (FCR), survival rate, and protein efficiency ratio (PER). The results of the orthogonal contrast analysis of all groups against each other are shown in Table S3 in Supplementary Materials. The carrots, which were available to the larvae as a water source, were consumed completely, but were not included in the following calculations. The individual weight gains of larvae ranged from 23.7 to 106.0 mg per larva. The highest LWGpL (106.0 ± 0.0 mg) was achieved by the control group of pure wheat bran. In all groups, it was observed that a decreasing protein content in the diet, due to a higher proportion of wheat bran, resulted in better larval growth. Comparing pea and rice protein, weight gain was linearly higher (*p* < 0.001) when fed rice protein. However, the addition of sweet lupine and potato flakes led to growth comparable (*p* = 0.063) with rice protein. Likewise, a similar trend was noticed in the SGR. Linear regression analysis showed significant correlation between the protein content of the diets and the individual larval weight (R^2^ = 0.572), as it was negatively influenced by higher dietary protein content. PPF80 showed the highest FCR (3.7 ± 0.9) among all groups, while SLF40 and RPF20 had a very low FCR (1.8–2.1) and were the most efficient diets. In contrast, when the protein efficiency ratio was taken into consideration, the feeding groups with pure wheat bran, potato flakes, sweet lupine flour (SLF20), and rice protein flour (RPF20) had the highest efficiency in terms of dietary protein content utilization with significant linear and quadratic contrast (*p* < 0.01). However, the contrast analysis showed significant linear and quadratic effects (*p* < 0.01) in the groups PPF80, PPF60, and PPF40 too, indicating that these groups had the poorest protein utilization efficiency. Low mortality of 2% and 4% was observed in the RPF80 and RPF60 groups, respectively. The remaining groups showed a survival rate of 100%.

### 3.2. Proximate Composition of Tenebrio molitor Larvae

The moisture, protein, and fat contents of mealworm larvae are presented in Table 6. The results of the orthogonal contrast analysis of the individual groups are shown in Table S4 in Supplementary Materials. Moisture content showed a wide variation (57.6–80.3%), as well as protein (36.3–74.1%) and fat content (20.3–48.6%). At the beginning of the experiment, the larvae recorded the lowest water content (57.6 ± 1.6%), whereas significant linear and quadratic (*p* < 0.001) increases in water content were observed at the end of the feeding trial. Both groups fed with a high proportion of pea protein (PPF80 and PPF60) had the highest respective water content (80.0 and 80.3%). Nevertheless, the protein content was high (74.1 and 74.0%) in the groups (PPF80 and PPF60) fed with a large amount of pea protein, respectively. Likewise, the orthogonal contrast analysis showed that a large proportion of rice protein (PPF80) had a linear and quadratic increase (*p* < 0.001) of the protein content (70.9 ± 0.1%). Nevertheless, it resulted in a significant linear (*p* < 0.001) and quadratic (*p* < 0.01) decrease in the fat content (20.3–22.8%). The proportionate feeding of cassava flour in the group CF10 resulted in the lowest protein content (36.3 ± 0.0%) and highest fat content (48.6 ± 0.5%). 

Linear regression analysis (Table 7) showed a significant correlation between the dietary protein content and the protein content of the larvae (R^2^ = 0.573). A weak correlation was also demonstrated with respect to the larval fat content (R^2^ = 0.253). The carbohydrate content of the diets significantly affected the larval protein (R^2^ = 0.432) and fat (R^2^ = 0.332) content. However, regression analysis showed no correlation between the dietary fat (R^2^ ≤ 0.114), fiber (R^2^ ≤ 0.143), and ash (R^2^ ≤ 0.073) content on larval protein and fat content.

### 3.3. Amino Acid Composition of Tenebrio molitor Larvae

The amino acid content of mealworm larvae is presented in Table 8. A total of 14 amino acids, including alanine (Ala), arginine (Arg), asparagine (Asp), glutamic acid (Glu), glycine (Gly), histidine (His), isoleucine (Ile), leucine (Leu), lysine (Lys), phenylalanine (Phe), serine (Ser), threonine (Thr), tyrosine (Tyr), and valine (Val), could be detected. A wide range of the amino acid content of the larvae (19.0–51.7%) could be determined. Ala, Asp, Gly, Leu, and Tyr are the most common amino acids in *Tenebrio molitor* larvae, whereas His and Phe are the least abundant. However, feeding cassava flour also resulted in the highest levels of Leu (10.9 ± 0.1%), Phe (8.2 ± 0.0%), and Ser (6.6 ± 0.0%) in the larvae as compared to the other feeding groups. In relation to the supplemented feeding groups, 14 amino acids were detected in the control group with pure wheat bran. In the groups fed with pea protein, an increased occurrence of amino acids with increasing dietary protein content was recorded. By contrast, the reverse could be observed in the groups supplemented with rice protein, where most amino acids showed higher content with decreasing dietary protein content. The total essential amino acid contents of mealworm larvae were calculated by adding the content of all essential amino acids, including His, Ile, Leu, Lys, Phe, Thr, Val, and Arg. The content of essential amino acids also showed a considerable range (10.8–30.4%). The highest levels of essential amino acids were analyzed in the larvae of the group CF10 (30.4 ± 0.2%), whereas the lowest concentration of essential amino acids (10.8–16.6%) were determined in the larvae of all PPF groups fed with pea protein flour.

## 4. Discussion

Our results and several previous studies have shown that *Tenebrio molitor* growth performance is directly influenced by feed composition, and specifically protein content. Rumbos et al. (2020) showed that specific amylaceous commodities (e.g., wheat bran, zea flour, durum wheat flour, and white flour) with protein contents between 11.1 and 14.2%, as well as two compound diets (milk-based feed and egg-layer hen feed) with protein contents of 36.7 and 16.3%, respectively, produced the highest larval biomass increase [41]. However, the total larval weight produced by the different legume flours with protein contents ranging from 22.9 to 42.4% was low [41]. In our study, an increasing amount of pea protein flour also reduced larval growth performance; feeding sweet lupin flour, by contrast, resulted in a comparable specific growth rate to pure wheat bran with no significant differences (*p* > 0.05, one-way ANOVA and contrast analysis). According to Oonincx et al. (2015), the quality of the diet had a significant influence on the food utilization parameters of mealworm larvae fed low- (12.9–14.4%) and high-protein (21.9–22.9%) diets [11]. In this study, FCRs ranging from 1.8 to 3.7 were comparable to different quality diets in other studies [11,32]. The highest FCR (3.7 ± 0.9) was calculated in larvae fed with pure pea protein flour (PPF80), but was not significantly different (*p* > 0.05, one-way ANOVA and contrast analysis) from the FCR (2.8 ± 0.0) of group RPF80, where larvae were fed with pure rice protein flour. However, as the protein content of the substrate decreased, the FCR improved. A number of insects, including *Tenebrio molitor*, have been observed to use ingested protein less efficiently once they reached a certain requirement for body growth [42]. Higher protein diets were found to have higher conversion efficiency [32], which is not comparable to our study. The diets with the highest protein content (60–80%), e.g., PPF80 and PPF60, as well as RPF80 and RPF60, obtained the lowest PER (0.6–1.2). Orthogonal contrast analysis showed significant linear and quadratic contrasts (*p* < 0.001) of the groups PPF80, PPF60, and PPF40 when comparing these with all other groups, resulting in the poorest protein efficiency ratio. However, there were differences in PER in the groups with the same protein content but a different protein source (rice and pea), as feeding pea protein flour resulted in significantly (*p* < 0.01) lower PER (0.6–1.8) than feeding rice protein (1.1–2.3). Similar results were presented by Zhang et al. (2019), who observed a low PER value (1.23) when they fed soy bean meal with high protein (43.18%) and amino acid (2.708 mg/g protein) content to mealworm larvae [43]. The highest PER (3.6 ± 0.2) with significant linear and quadratic effects (*p* < 0.001) was reached by the control group fed with wheat bran, which is in line with the results of Ochoa Sanabria et al. (2019), who reported values of 3.1 to 3.7 for PER when mealworm larvae were fed with wheat [44]. Consequently, although insects generally grow faster when fed high protein diets, the protein economy of a production facility may be less efficient. Comparatively, lipids are stored directly proportional to the amount of energy they contain from lipids and carbohydrates [45]. Protein is utilized most efficiently for tissue growth at the highest possible ratio, while carbohydrates and lipids are utilized for energy, as this produces the most rapid increase in protein body mass [26,45]. Rho and Lee (2016) declared that, for *Tenebrio molitor* larvae, the optimal protein to carbohydrate ratio lies around 1:1 for optimal growth [46]. Generally, insects grow faster when they consume diets high in protein [10]. However, it has been observed by Rumbos et al. (2020) that almost all legume flours tested inhibit the growth of *Tenebrio molitor* larvae, even though they contain the highest amount of protein (22.9–42.4%) of the substrates tested [41]. Thus, it becomes clear that substrate protein content, while relevant, is not the only factor to consider in determining whether a substrate is suitable for the growth of mealworm larvae. In this study, larval weight gain and growth rate decreased as the dietary protein content increased from 20 to 80% on a fresh weight basis in the groups fed with pea and rice protein. Comparing pea and rice protein, orthogonal contrast analysis showed significant (*p* < 0.001) linear and quadratic higher weight gain and protein conversion ratio when fed rice protein. Feeding pea protein flour resulted in higher FCR compared to rice protein flour, but no significant differences (*p* > 0.05, one-way ANOVA and contrast analysis) were found among the pea and rice protein groups with the same protein content. The supplementation of pea protein resulted in higher dietary fat content and lower dietary carbohydrate content, which can reduce larval growth [47]. In plants, fiber is typically composed of cellulose, lignin, and hemicelluloses and the content of these components varies among vegetable species [48]. Cereals and other members of the monocot class, such as wheat and rice, contain fiber high in hemicellulose and are moderately high in lignin, while legumes, such as pea, are high in lignin and low in hemicellulose [48]. Therefore, not only is the carbohydrate or crude fiber content important for efficient larval growth, but also the composition and type of plant fiber. The better growth of the larvae fed with rice protein flour may be due to the fact that they were able to utilize the type of rice fiber better than those of pea. However, the type of carbohydrates (e.g., starch) can have a significant impact on larval growth too. Tenebrionidae have a higher digestion resistance to potato starch than to starch of wheat or maize [49,50], which may explain the higher growth rates and utilization efficiency of the larvae fed with wheat bran compared to those fed with a supplementation. Nevertheless, for exact predictions, the carbohydrate and fiber composition of each substrate and diet must be clarified, which can be addressed in more detail in following studies.

As demonstrated by linear regression analysis (R^2^ = 0.572), higher dietary protein content was negatively correlated with individual larval weight. Contrary to our results, Rumbos et al. (2020) observed no correlation (R^2^ = 0.01) between the protein content of the substrates and the larval weight [41]. Nevertheless, we examined a wider range of protein contents (10.1–80.0%) than they did, so dietary protein content varied more, which could be the reason for the positive correlation. In accordance with our results, Morales-Ramos et al. (2020) also tested many different substrates with different nutritional content and found significant effects on live biomass gain (R^2^ = 0.7) related to the consumption of relevant ingredients [51]. Mancini et al. (2019) reported that the crude protein content of Tenebrio molitor larvae were affected by the diet, as the low content of crude protein in cookies (6.55%) negatively influenced the growth and nutritional composition of the larvae with the result of the lowest weight (87 mg per larva) and larval protein content (37.31% dry matter) [35]. The nutrient composition of the larvae, especially the protein content, varied in the study of Zhang et al. (2019), who reported high larval protein contents (>70%) when larvae were fed with substrates varying in protein content (4–43%). Slower growth rates were observed when feeding mushroom spent corn stover [43], probably depending on the low protein content (4%) in the diet, as larvae tend to consume less and gain less weight on nutrient-imbalanced diets [52]. Therefore, an increase in substrate protein content may not be necessary to improve larval growth. However, dietary amino acids greatly affect the life cycle of mealworm larvae too, contributing directly to larval growth, weight gain, and survival. A diet supplemented with 10% yeast and 90% whole ground wheat with a protein content of 11% during a period of 4 weeks enhanced larval growth, resulting in 45.5–55.6 mg weight gain per larva compared to 2.3–2.9 mg on a protein-free diet [53]. In an ideal diet for *Tenebrio molitor* larvae, amino acids should be provided at levels similar to those in larvae tissues, except for phenylalanine, which should be offered at 50% of the body mass concentration, and of tryptophan and threonine, which should be fed at twice the concentrations found in larval tissues [53]. It has been concluded that *Tenebrio molitor* larvae require a diet containing the same 10 amino acids that are essential for growth in other vertebrates, while cystine, aspartic acid, alanine, and proline are semiessential, and tyrosine, glutamic acid, serine, and possibly glycine are unessential for insect growth [28]. In our experiment, we were able to detect a total of 14 amino acids, including all essential amino acids (except tryptophan and methionine) for *Tenebrio molitor* larvae, with a comparable range of amino acid content of other studies [23,54,55]. The amino acid values of this study differed depending on diet. The groups with the highest contents of amino acids were CF10 (51.7 ± 0.5%), fed with cassava flour and wheat bran, followed by SLF20 (47.9 ± 0.0%) and SLF40 (44.2 ± 0.3%), which were fed with sweet lupine flour and wheat bran. Consequently, feeding a mixture of wheat bran and cassava and sweet lupine flour, respectively, resulted in higher amino acid concentrations compared to the control group WB (only wheat bran), which had a lower amino acid content (38.8 ± 0.0%). According to Adámková et al. (2020), it was also found that the amino acid content of mealworm larvae after feeding a mixture of wheat bran and lentil flour resulted in higher amino acid content compared with the control group which was fed pure wheat bran [56]. However, this research also demonstrated that substrates, such as cassava and sweet lupine flour, with low protein and amino acid content cause larvae to accumulate amino acids in the larval body. Therefore, it is not necessary to feed high amounts of surplus amino acids since the larvae will excrete these amino acids [56]. The results of this study also indicate that the formation of amino acids does not require a high protein content in the diet. This is because nonessential amino acids are synthesized from intermediary products of glucose degradation, while essential amino acids have to be ingested with food or are formed by symbionts [57,58,59]. However, it should be mentioned that the major influencing factor is limiting the essential amino acids, which is important for the prevailing contribution of the improved growth performance and of changes in nutritive composition of *Tenebrio molitor* larvae, as shown in the study by John et al. (1979) [53].

Nevertheless, as mentioned before, the amino acid and protein content is not only crucial for larval growth, but also for the changes in the larval composition. The protein content of the larvae increased with the protein content of the diet. A similar trend could be observed in a feeding study with *Tenebrio molitor* larvae from Fondevila and Fondevila (2022) [60], where substrate mixtures based on barley straw and wheat grain with increasing contents of soybean meal were fed. This also led to greater larval protein content with higher dietary protein content. In the present work, mealworm larvae had a protein content between 36.3 and 74.1% on a dry matter basis. *Tenebrio molitor* larvae reared on commercial oat feed and wheat germ showed protein levels between 63.3 and 68.9% by dry weight, which is within the range of this research. Furthermore, as previously reported, edible insects contain high crude protein levels, with values of 40.0 to 75.0% of dry weight [61,62,63], and are consistent with our study. The varying nutritional composition of the larvae can be reached either by gut loading, which involves feeding a substrate or diet with increased concentrations of a specific nutrient for a short period of time prior to insect harvesting (this will increase the concentration of this nutrient in the insect digestive tract) [64,65], or by altering the body composition of the insect after feeding a specific substrate/diet over a long period of time [11,32]. In the present work, mealworm larvae were starved for 24 h prior to harvesting. Therefore, it is likely that larval digestive tract composition had a minimal impact on the nutritional composition of the larvae and the results of the nutritional content are reflected in the insect body composition. However, it is also important to mention that a significant influence on the protein content of *Tenebrio molitor* larvae can be attributed to the feed quality rather than the quantity [41]. As observed in our study, insects will have a higher protein content if their feed contains more protein. Regression analysis indicated a correlation between dietary protein content and the protein (R^2^ = 0.573) and fat (R^2^ = 0.253) content of the larvae. These findings were in line with previous research of Jajić et al. (2022), who observed a correlation of main nutrients in the substrates and their content in the larvae [66]. However, in their study, the correlation in terms of dietary and larval protein content was weak (R^2^ = 0.3169) [66]. The regression coefficient for the comparison of dietary protein and larval fat content (R^2^ = 0.2508) was comparable to ours [66]. Rumbos et al. (2020) also reported a weak relationship between protein content of the substrates and the protein content of the larvae (R^2^ = 0.36) [41], and Melis et al. (2019) showed quite similar crude protein contents in both rearing substrates (19.57–22.45%) and the related protein contents of mealworm larvae (13.35–14.78% fresh weight) [67]. However, there have also been cases when this rule has not been observed. In the research study of Bordiean et al. (2022), mealworm larvae consumed some diets where the protein content was almost the same (20.2–20.4%) but showed differences in protein content (50.9–53.4%) [68]. Accordingly, even feeds with poor or low protein quality can be used, resulting in insects with high protein concentrations. A similar scenario was observed in another study, where different substrates with high (43.2%) and low (3.9%) protein contents were fed to mealworm larvae, which had a comparable protein content (69.9–75.3%) at the end of the experiment [69]. Nevertheless, protein content is usually overestimated due to the presence of nonprotein nitrogen from chitin, which is derived from nitrogen content multiplied by a conversion factor of 6.25. Chitin was not analyzed and subtracted from the protein content in this study, so the protein content of mealworm larvae includes chitin and other nitrogen-containing components too. Furthermore, a high protein content in the larvae can also indicate that the larvae are undersupplied and unable to accumulate their fat reserves.

The effect of diet on the protein content of *Tenebrio molitor* larvae has generally been considered different in various studies, whereas diet appears to significantly affect the lipid content [10,32]. Fat is the second most important component of insect nutrient composition [70] and varies greatly between 13.4 and 33.4% on a dry matter basis [62]. There are many factors that influence insect fat content, including sex, species, reproduction stages, diet, and habitat [71]. In the present research, the highest fat content, with significant linear (*p* < 0.001) and quadratic (*p* < 0.01) contrasts, was examined in the larvae from groups CF10 (48.6 ± 0.5%) and PF10 (47.8 ± 0.4%), which were fed with potato flakes and cassava flour, respectively. Both diets have a high carbohydrate content (60.2–66.9%), which can cause the fat content of the larvae to increase, since fatty acids, from 12 to 18 carbons, can be biosynthesized de novo by various insect species [72] and are produced from dietary carbohydrates regulated by fatty acid synthase (FAS) and acetyl-CoA carboxylase (ACC) [73]. It can be concluded that the dietary fat content, which was very low (1.8–3.0%) in both substrates, has a lesser influence on the larval fat content than the dietary carbohydrate content. In our study, the dietary carbohydrate content affected the larval protein (R^2^ = 0.432) and fat (R^2^ = 0.332) content. These results were also comparable to Jajić et al. (2022), although their study showed an even higher correlation between the carbohydrate content of the substrate and the protein (R^2^ = 0.5711) and fat (R^2^ = 0.4458) content of the larvae [66]. Rho and Lee (2014) suggested that a diet containing a low protein to carbohydrate ratio (0:42 and 7:35) results in higher lipid concentrations in mealworms [42]. This effect has also been demonstrated in another study [74], whereas the fat content of *Tenebrio molitor* was higher on carbohydrate-rich diets. A diet rich in carbohydrates and lipids can contribute to produce insects that are lipid-rich, while a diet rich in protein enables insects to be lean [45,72]. *Tenebrio molitor* larvae need a high content of carbohydrates in their diet, with an optimal range of 80–85% [25]. Davis (1974) observed less growth of mealworms when fed sucrose, starch, or lactose instead of glucose in a diet containing amino acid mixtures [74], although Fraenkel (1950) reported no significant differences [25]. The maximum growth rate was observed when the diet contained a minimum of 50% carbohydrates and more than 15% protein [75]. It has been reported that consumption of diets consisting of plant materials, yeast, and *Tenebrio molitor* excreta results in twofold higher protein levels and five- to sixfold increased body fat levels than those obtained from the substrate, with significant reductions in fiber and carbohydrate content [7].

However, the moisture content, too, of the larvae can have an effect on the nutrient composition of the larvae, since changes in moisture may also cause the other nutrients (e.g., protein and fat) to vary in content. The moisture content of *Tenebrio molitor* larvae presented here is comparable to other studies [12,41,76]. Several factors (e.g., water source, diet, and relative humidity) can influence the moisture content of mealworm larvae [12,76,77]. *Tenebrio molitor* larvae showed a significant linear (*p* < 0.001) and quadratic (*p* <0.001) higher moisture content in the groups PPF80 and PPF60, which were fed with a high proportion of pea protein flour, because this substrate was highly hygroscopic. Some studies have demonstrated that protein hydrolysates often have hygroscopic properties [78,79]. The pea protein flour was able to accumulate water during the experimental period, which was consumed by the larvae during substrate ingestion. In our previous publication, we were able to show that the change in the dietary moisture content has a significant effect on the nutrient composition of the larvae [76]. As such, it is important to measure the moisture content of the larvae when taking a closer look at their composition and nutritional changes.

## 5. Conclusions

In our study, we were able to show that dietary protein content is not the only determinant of larval growth and changes in nutritional composition. As a result, the fat and carbohydrate content of the diet also has a significant influence. Furthermore, a high dietary protein and amino acid content does not simultaneously guarantee a high protein and amino acid content in the larvae since carbohydrates are also partly responsible here. A protein content higher than 20% had a negative effect on growth and feed conversion ratio. In addition, it was shown that a high content of dietary amino acids, especially essential amino acids, did not lead to an accumulation of these amino acids in the larva. An important result of this work is that it became clear that the protein source was essential for growth and the influence on nutrient composition, as, for e.g., when were larvae fed with rice protein and pea protein with the same protein content, rice protein led to better growth performance and feed conversion efficiency, with a higher protein efficiency ratio and significantly different larval protein content. The results of this research may lead to the production of improved formulations of artificial diets for *Tenebrio molitor* larvae in the future. Nevertheless, further research is necessary to understand the complex metabolic processes and the dietary requirements of *Tenebrio molitor* larvae.

## Figures and Tables

**Table 1:** Amount (%) of the individual substrates to create experimental diets for *Tenebrio molitor* larvae.

Group	Substrate Amount (%)					
	Pea Protein Flour (PPF)	Rice Protein Flour (RPF)	Sweet Lupine Flour (SLF)	Cassava Flour (CF)	Potato Flakes (PF)	Wheat Bran (WB)
PPF80	100.0	-	-	-	-	-
PPF60	69.3	-	-	-	-	30.7
PPF40	38.6	-	-	-	-	61.4
PPF20	7.9	-	-	-	-	92.1
RPF80	-	100.0	-	-	-	-
RPF60	-	69.3	-	-	-	30.7
RPF40	-	38.6	-	-	-	61.4
RPF20	-	7.9	-	-	-	92.1
SLF40	-	-	89.4	-	-	10.6
SLF20	-	-	18.2	-	-	81.8
CF10	-	-	-	37.0	-	63.0
PF10	-	-	-	-	70.1	29.9
WB (Control)	-	-	-	-	-	100.0

PPF80: pea protein flour (80% protein); PPF60: pea protein flour and wheat bran (60% protein); PPF40: pea protein flour and wheat bran (40% protein); PPF20: pea protein flour and wheat bran (20% protein); RPF80: rice protein flour (80% protein); RPF60: rice protein flour and wheat bran (60% protein); RPF40: rice protein flour and wheat bran (40% protein); RPF20: rice protein flour and wheat bran (20% protein); SLF40: sweet lupine flour and wheat bran (40% protein); SLF20: sweet lupine flour and wheat bran (20% protein); CF10: cassava flour and wheat bran (10% protein); PF10: potato flakes and wheat bran (10% protein); WB: wheat bran (control).

**Table 2:** Nutritional composition of the diets on a fresh weight (FW) basis (%) used for *Tenebrio molitor* larvae feeding.

Group	Moisture (%)	Crude Protein * (% FW)	Crude Fat (% FW)	Crude Carbohydrate (% FW)	Crude Fiber (% FW)	Crude Ash (% FW)
PPF80	2.7	80.0	8.0	4.9	4.2	0.2
PPF60	5.6	60.0	7.0	17.2	8.3	1.9
PPF40	8.4	40.0	6.0	29.5	12.5	3.6
PPF20	11.3	20.0	5.0	41.8	16.6	5.3
RPF80	3.9	80.0	2.9	9.6	3.3	0.3
RPF60	6.4	60.0	3.5	20.5	5.4	4.2
RPF40	8.9	40.0	4.0	31.3	10.9	4.9
RPF20	11.4	20.0	4.6	42.2	16.3	5.5
SLF40	6.3	40.0	11.2	13.7	26.9	1.9
SLF20	10.8	20.0	6.1	38.6	19.6	4.9
CF10	9.6	10.1	3.0	60.2	13.4	3.7
PF10	10.0	10.3	1.8	66.9	8.5	2.5
WB (Control)	12.0	14.9	4.7	45.0	17.7	5.7

PPF80: pea protein flour (80% protein); PPF60: pea protein flour and wheat bran (60% protein); PPF40: pea protein flour and wheat bran (40% protein); PPF20: pea protein flour and wheat bran (20% protein); RPF80: rice protein flour (80% protein); RPF60: rice protein flour and wheat bran (60% protein); RPF40: rice protein flour and wheat bran (40% protein); RPF20: rice protein flour and wheat bran (20% protein); SLF40: sweet lupine flour and wheat bran (40% protein); SLF20: sweet lupine flour and wheat bran (20% protein); CF10: cassava flour and wheat bran (10% protein); PF10: potato flakes and wheat bran (10% protein); WB: wheat bran (control), * protein content was calculated with the conversion factor of 6.25.

**Table 3:** Amino acid content (g/100 g of protein) of the diets used for *Tenebrio molitor* larvae feeding. Data are presented as mean ± standard deviation, *n* = 2.

Amino Acid (% DM)	Group												
	PPF80	PPF60	PPF40	PPF20	RPF80	RPF60	RPF40	RPF20	SLF40	SLF20	CF10	PF10	WB
Ala	4.1 ± 0.08	3.1 ± 0.03	2.0 ± 0.07	0.9 ± 0.07	4.0 ± 0.03	2.9 ± 0.08	1.9 ± 0.05	0.9 ± 0.05	1.1 ± 0.03	0.9 ± 0.04	0.3 ± 0.04	0.5 ± 0.05	0.6 ± 0.05
Arg	6.4 ± 0.04	4.6 ± 0.02	3.0 ± 0.08	1.2 ± 0.04	8.2 ± 0.02	5.9 ± 0.07	3.7 ± 0.03	1.4 ± 0.04	3.5 ± 0.02	2.4 ± 0.05	0.5 ± 0.05	0.6 ± 0.03	0.8 ± 0.06
Asp	7.1 ± 0.04	5.2 ± 0.06	3.4 ± 0.04	1.5 ± 0.03	11.3 ± 0.04	8.1 ± 0.08	5.0 ± 0.05	1.8 ± 0.02	3.3 ± 0.05	2.4 ± 0.04	0.5 ± 0.08	1.3 ± 0.07	1.0 ± 0.04
Glu	14.9 ± 0.03	11.4 ± 0.07	7.9 ± 0.03	4.4 ± 0.06	18.2 ± 0.03	13.6 ± 0.05	9.2 ± 0.07	4.7 ± 0.07	7.8 ± 0.07	6.1 ± 0.05	1.8 ± 0.03	2.4 ± 0.09	3.5 ± 0.08
Gly	3.4 ± 0.07	2.5 ± 0.03	1.7 ± 0.05	0.9 ± 0.03	3.7 ± 0.06	2.7 ± 0.05	1.8 ± 0.03	0.9 ± 0.06	1.3 ± 0.04	1.0 ± 0.05	1.8 ± 0.02	0.5 ± 0.07	0.6 ± 0.08
His	1.7 ± 0.08	1.3 ± 0.04	0.9 ± 0.08	0.4 ± 0.06	2.1 ± 0.04	1.6 ± 0.04	1.0 ± 0.05	0.4 ± 0.04	0.4 ± 0.07	0.4 ± 0.08	0.2 ± 0.06	0.2 ± 0.05	0.3 ± 0.07
Ile	3.2 ± 0.05	2.3 ± 0.07	1.5 ± 0.09	0.6 ± 0.07	4.5 ± 0.02	3.2 ± 0.07	2.0 ± 0.03	0.8 ± 0.08	1.3 ± 0.04	1.0 ± 0.05	0.2 ± 0.06	0.4 ± 0.03	0.4 ± 0.05
Leu	6.2 ± 0.03	4.6 ± 0.08	3.0 ± 0.03	1.4 ± 0.07	8.0 ± 0.07	5.8 ± 0.03	3.7 ± 0.05	1.5 ± 0.09	2.4 ± 0.08	1.8 ± 0.03	0.5 ± 0.06	0.8 ± 0.05	1.0 ± 0.05
Lys	2.4 ± 0.04	1.7 ± 0.03	1.1 ± 0.02	0.5 ± 0.08	6.3 ± 0.09	4.5 ± 0.03	2.7 ± 0.06	0.8 ± 0.09	0.9 ± 0.04	0.7 ± 0.06	0.2 ± 0.08	0.3 ± 0.07	0.3 ± 0.08
Phe	4.1 ± 0.05	3.0 ± 0.04	1.9 ± 0.05	0.8 ± 0.04	5.3 ±0.04	3.9 ± 0.06	2.4 ± 0.07	0.9 ± 0.04	1.2 ± 0.07	0.9 ± 0.08	0.3 ± 0.09	0.5 ± 0.09	0.6 ± 0.08
Ser	3.9 ± 0.07	2.9 ± 0.05	1.9 ± 0.06	0.9 ± 0.03	4.9 ± 0.07	3.6 ± 0.07	2.3 ± 0.09	1.0 ± 0.04	1.8 ± 0.08	1.3 ± 0.04	0.4 ± 0.07	0.5 ± 0.06	0.7 ± 0.06
Thr	2.7 ± 0.03	2.0 ± 0.06	1.3 ± 0.03	0.5 ± 0.07	3.1 ± 0.01	2.3 ± 0.03	1.4 ± 0.05	0.6 ± 0.06	1.1 ± 0.05	0.8 ± 0.02	0.2 ± 0.03	0.4 ± 0.05	0.3 ± 0.04
Tyr	4.3 ± 0.05	3.1 ± 0.02	1.9 ± 0.08	0.7 ± 0.04	4.0 ± 0.06	2.9 ± 0.02	1.8 ± 0.03	0.7 ± 0.03	1.4 ± 0.03	1.0 ± 0.01	0.2 ± 0.02	0.4 ± 0.03	0.4 ± 0.07
Val	4.6 ± 0.07	3.4 ± 0.04	2.2 ± 0.05	1.0 ± 0.07	4.9 ± 0.04	3.6 ± 0.06	2.3 ± 0.07	1.0 ± 0.07	1.3 ± 0.07	1.0 ± 0.07	0.3 ± 0.05	0.5 ± 0.05	0.6 ± 0.03
Total AAs	69.0 ± 0.07	51.1 ± 0.06	33.7 ± 0.07	15.7 ± 0.06	88.5 ± 0.05	64.6 ± 0.06	41.2 ± 0.05	17.4 ± 0.07	28.8 ± 0.07	21.7 ± 0.05	6.1 ± 0.07	6.9 ± 0.06	11.1 ± 0.07
Essential AAs	31.3 ± 0.06	22.9 ± 0.05	14.9 ± 0.06	6.4 ± 0.05	42.4 ± 0.04	30.6 ± 0.04	19.2 ± 0.06	7.4 ± 0.06	12.1 ± 0.06	9.0 ± 0.05	2.4 ± 0.05	3.7 ± 0.05	4.3 ± 0.06

n. d.: not detected; Ala: alanine; Arg: arginine; Asp: aspartic acid; Glu: glutamic acid; Gly: glycine; His: histidine; Ile: isoleucine; Leu: leucine; Lys: lysine; Phe: phenylalanine; Ser: serine; Thr: threonine; Tyr: tyrosine; Val: valine; Total AAs: total amino acids; Essential AAs: essential amino acids; PPF80: pea protein flour (80% protein); PPF60: pea protein flour and wheat bran (60% protein); PPF40: pea protein flour and wheat bran (40% protein); PPF20: pea protein flour and wheat bran (20% protein); RPF80: rice protein flour (80% protein); RPF60: rice protein flour and wheat bran (60% protein); RPF40: rice protein flour and wheat bran (40% protein); RPF20: rice protein flour and wheat bran (20% protein); SLF40: sweet lupine flour and wheat bran (40% protein); SLF20: sweet lupine flour and wheat bran (20% protein); CF10: cassava flour and wheat bran (10% protein); PF10: potato flakes and wheat bran (10% protein); WB: wheat bran (control).

**Table 4:** Flow gradient program.

Time (min)	Eluent A (%)	Eluent B (%)	Flow (mL/min)
0	90	10	0.4
40	60	40	0.4
65	0	100	0.4
68	0	100	0.4
75	90	10	0.4
85	90	10	0.4

**Table 5:** Growth performance, feed and protein utilization efficiency, and survival rate of *Tenebrio molitor* larvae of different feeding groups. Values are given as mean ± standard deviation, *n* = 5.

Group	LWGpL (mg)	SGR (% per Day)	FCR (-)	Survival Rate (%)	PER (-)
PPF80	23.7 ± 5.9 ^h^	4.9 ± 0.5 ^d^	3.7 ± 0.9 ^a^	100.0 ± 0.0 ^a^	0.6 ± 0.1 ^d^
PPF60	42.5 ± 0.8 ^g^	6.0 ± 0.1 ^c^	2.8 ± 0.1 ^a,b^	100.0 ± 0.0 ^a^	0.7 ± 0.1 ^d^
PPF40	54.7 ± 1.3 ^f^	6.6 ± 0.2 ^c^	2.4 ± 0.1 ^b^	100.0 ± 0.0 ^a^	0.9 ± 0.2 ^d^
PPF20	71.1 ± 1.6 ^d^	7.4 ± 0.1 ^b^	2.1 ± 0.0 ^b,c^	99.0 ± 0.1 ^a^	1.8 ± 0.3 ^c^
RPF80	52.2 ± 5.7 ^f^	5.5 ± 0.7 ^c,d^	2.8 ± 0.0 ^ab^	96.0 ± 0.3 ^b^	1.1 ± 0.2 ^c^
RPF60	70.3 ± 3.4 ^d^	7.3 ± 0.1 ^b^	2.3 ± 0.0 ^b^	98.0 ± 0.1 ^a^	1.2 ± 0.1 ^c^
RPF40	82.3 ± 1.8 ^c^	7.7 ± 0.1 ^a^	2.2 ± 0.0 ^b^	100.0 ± 0.0 ^a^	1.4 ± 0.1 ^c^
RPF20	91.5 ± 1.2 ^b^	7.9 ± 0.1 ^a^	2.0 ± 0.0 ^c^	100.0 ± 0.0 ^a^	2.3 ± 0.2 ^b^
SLF40	85.9 ± 2.4 ^c^	7.9 ± 0.1 ^a^	1.8 ± 0.0 ^c^	100.0 ± 0.0 ^a^	1.5 ± 0.1 ^c^
SLF20	90.4 ± 1.1 ^b^	8.0 ± 0.0 ^a^	2.2 ± 0.0 ^b,c^	100.0 ± 0.0 ^a^	2.5 ± 0.3 ^b^
CF10	62.0 ± 2.0 ^e^	7.0 ± 0.1 ^b,c^	3.2 ± 0.1 ^a^	100.0 ± 0.0 ^a^	1.5 ± 0.4 ^c^
PF10	90.0 ± 1.0 ^b^	8.0 ± 0.0 ^a^	2.2 ± 0.0 ^b,c^	100.0 ± 0.0 ^a^	2.7 ± 0.3 ^b^
WB (Control)	106.0 ± 0.9 ^a^	8.1 ± 0.0 ^a^	2.3 ± 0.0 ^b^	100.0 ± 0.0 ^a^	3.6 ± 0.2 ^a^
*p*-value linear	<0.001	<0.001	<0.001	0.041	<0.01
*p*-value quadratic	<0.001	<0.01	<0.01	0.043	<0.01

LWGpL: larval weight gain per larvae; SGR: specific growth rate; FCR: feed conversion ration; PER: protein efficiency ratio; PPF80: pea protein flour (80% protein); PPF60: pea protein flour and wheat bran (60% protein); PPF40: pea protein flour and wheat bran (40% protein); PPF20: pea protein flour and wheat bran (20% protein); RPF80: rice protein flour (80% protein); RPF60: rice protein flour and wheat bran (60% protein); RPF40: rice protein flour and wheat bran (40% protein); RPF20: rice protein flour and wheat bran (20% protein); SLF40: sweet lupine flour and wheat bran (40% protein); SLF20: sweet lupine flour and wheat bran (20% protein); CF10: cassava flour and wheat bran (10% protein); PF10: potato flakes and wheat bran (10% protein); WB: wheat bran (control). ^a–h^ Different superscripts in the same column denote significant differences, *p* < 0.05.

**Table 6:** Nutritional composition on a dry matter (DM) basis (%) of *Tenebrio molitor* larvae. Values are given as mean ± standard deviation, *n* = 3.

Group	Moisture (%)	Crude Protein (% DM)	Crude Fat (% DM)
PPF80	80.3 ± 0.1 ^a^	74.1 ± 0.2 ^a^	20.3 ± 0.3 ^e^
PPF60	80.0 ± 0.4 ^a,^	74.0 ± 0.0 ^a^	21.5 ± 0.7 ^e^
PPF40	67.1 ± 0.1 ^b^	51.1 ± 0.1 ^d^	31.3 ± 0.4 ^d^
PPF20	64.6 ± 0.1 ^c^	52.3 ± 0.2 ^d^	37.3 ± 0.5 ^c^
RPF80	68.4 ± 1.6 ^b^	70.9 ± 0.1 ^b^	22.8 ± 0.4 ^e^
RPF60	63.4 ± 0.1 ^b^	60.4 ± 0.2 ^c^	29.5 ± 0.3 ^d^
RPF40	64.6 ± 0.3 ^c^	59.6 ± 0.0 ^c^	29.9 ± 0.2 ^d^
RPF20	64.2 ± 0.3 ^c^	57.5 ± 0.1 ^c^	34.4 ± 0.4 ^c^
SLF40	58.8 ± 1.4 ^d^	48.8 ± 0.2 ^d^	37.6 ± 0.4 ^c^
SLF20	60.6 ± 0.6 ^d^	49.7 ± 0.3 ^d^	41.9 ± 0.3 ^b^
CF10	58.4 ± 0.4 ^d^	36.3 ± 0.0 ^e^	48.6 ± 0.5 ^a^
PF10	59.0 ± 0.2 ^d^	41.5 ± 0.1 ^e^	47.8 ± 0.4 ^a^
WB (Control)	68.6 ± 0.3 ^b^	60.5 ± 0.2 ^c^	35.4 ± 0.5 ^c^
Start	57.6 ± 1.6 ^d^	52.6 ± 0.3 ^d^	25.5 ± 1.3 ^d^
*p*-value linear	<0.001	<0.001	<0.001
*p*-value quadratic	<0.001	<0.001	<0.01

PPF80: pea protein flour (80% protein); PPF60: pea protein flour and wheat bran (60% protein); PPF40: pea protein flour and wheat bran (40% protein); PPF20: pea protein flour and wheat bran (20% protein); RPF80: rice protein flour (80% protein); RPF60: rice protein flour and wheat bran (60% protein); RPF40: rice protein flour and wheat bran (40% protein); RPF20: rice protein flour and wheat bran (20% protein); SLF40: sweet lupine flour and wheat bran (40% protein); SLF20: sweet lupine flour and wheat bran (20% protein); CF10: cassava flour and wheat bran (10% protein); PF10: potato flakes and wheat bran (10% protein); WB: wheat bran (control); Start: larvae at the beginning of the experiment. ^a–e^ Different superscripts in the same column denote significant differences, *p* < 0.05.

**Table 7:** Regression coefficients (R^2^) showing correlation of the diet composition with protein and fat contents of *Tenebrio molitor* larvae after linear regression analysis.

	Diet				
Larvae	Crude Protein	Crude Fat	Crude Carbohydrate	Crude Fiber	Crude Ash
Protein	0.573	0.114	0.432	0.143	0.073
Fat	0.253	0.033	0.332	0.136	0.032

**Table 8:** Amino acid content of *Tenebrio molitor* larvae of different feeding groups (g/100 g of protein). Data are presented as mean ± standard deviation, *n* = 2.

Amino Acid (% DM)	Group													
	PPF80	PPF60	PPF40	PPF20	RPF80	RPF60	RPF40	RPF20	SLF40	SLF20	CF10	PF10	WB	Start
Ala	5.5 ± 0.21	4.9 ± 0.03	3.2 ± 0.14	2.5 ± 0.03	4.3 ± 0.07	4.7 ± 0.03	5.0 ± 0.09	5.3 ± 0.05	5.2 ± 0.02	5.6 ± 0.03	4.3 ± 0.02	3.3 ± 0.05	3.9 ± 0.03	4.4 ± 0.03
Arg	2.7 ± 0.03	2.5 ± 0.04	1.9 ± 0.03	1.8 ± 0.04	3.1 ± 0.06	3.5 ± 0.05	3.7 ± 0.03	3.9 ± 0.09	3.3 ± 0.12	3.8 ± 0.05	2.3 ± 0.12	2.3 ± 0.04	2.2 ± 0.04	2.8 ± 0.04
Asp	0.5 ± 0.04	0.7 ± 0.02	1.6 ± 0.02	1.8 ± 0.02	n. d.	n. d.	3.0 ± 0.22	3.2 ± 0.07	4.2 ± 0.04	4.3 ± 0.07	n. d.	n. d.	3.9 ± 0.02	n. d.
Glu	n. d.	n. d.	n. d.	n. d.	n. d.	n. d.	n. d.	n. d.	8.9 ± 0.01	8.9 ± 0.03	3.8 ± 0.32	n. d.	6.2 ± 0.05	n. d.
Gly	3.7 ± 0.14	2.8 ± 0.05	2.8 ± 0.13	1.7 ± 0.03	2.7 ± 0.07	2.5 ± 0.08	3.2 ± 0.02	3.4 ± 0.05	3.1 ± 0.21	3.5 ± 0.02	3.6 ± 0.11	2.5 ± 0.03	2.5 ± 0.06	1.3 ± 0.02
His	1.5 ± 0.13	1.3 ± 0.06	1.1 ± 0.04	1.0 ± 0.04	1.7 ± 0.05	1.2 ± 0.05	1.6 ± 0.02	1.7 ± 0.06	1.6 ± 0.05	2.0 ± 0.05	1.3 ± 0.03	1.3 ± 0.05	1.1 ± 0.07	1.6 ± 0.06
Ile	2.8 ± 0.12	2.5 ± 0.03	1.7 ± 0.05	1.5 ± 0.06	2.5 ± 0.03	2.4 ± 0.03	3.2 ± 0.06	3.3 ± 0.03	2.6 ± 0.07	2.7 ± 0.03	1.6 ± 0.02	1.9 ± 0.03	1.9 ± 0.03	2.3 ± 0.07
Leu	3.4 ± 0.16	2.8 ± 0.02	2.8 ± 0.04	2.2 ± 0.02	3.7 ± 0.06	3.6 ± 0.08	4.7 ± 0.02	4.7 ± 0.03	4.0 ± 0.04	4.3 ± 0.03	10.9 ± 0.10	2.8 ± 0.02	3.3 ± 0.02	3.8 ± 0.05
Lys	2.7 ± 0.19	2.6 ± 0.08	1.9 ± 0.02	1.6 ± 0.05	3.3 ± 0.08	3.3 ± 0.04	3.6 ± 0.07	3.7 ± 0.05	3.2 ± 0.03	3.2 ± 0.03	1.2 ± 0.04	2.7 ± 0.04	2.6 ± 0.03	2.7 ± 0.13
Phe	1.9 ± 0.04	1.4 ± 0.09	1.2 ± 0.04	1.5 ± 0.02	2.4 ± 0.03	2.3 ± 0.03	2.7 ± 0.05	2.8 ± 0.03	n. d.	n. d.	8.2 ± 0.04	1.3 ± 0.07	1.5 ± 0.05	2.1 ± 0.03
Ser	n. d.	n. d.	n. d.	n. d.	n. d.	n. d.	n. d.	n. d.	n. d.	2.9 ± 0.08	6.6 ± 0.03	n. d.	2.2 ± 0.07	n. d.
Thr	1.6 ± 0.05	1.5 ± 0.08	1.4 ± 0.05	1.2 ± 0.03	2.2 ± 0.03	2.4 ± 0.03	2.3 ± 0.08	2.4 ± 0.03	2.3 ± 0.04	2.3 ± 0.04	2.0 ± 0.02	2.0 ± 0.05	1.9 ± 0.04	6.4 ± 0.04
Tyr	2.4 ± 0.06	2.7 ±0.06	2.6 ± 0.07	2.2 ± 0.05	4.1 ± 0.06	4.6 ± 0.07	4.6 ± 0.06	4.7 ± 0.05	5.8 ± 0.04	4.4 ± 0.05	2.9 ± 0.01	2.3 ± 0.06	2.9 ± 0.05	4.5 ± 0.04
Val	n. d.	n. d.	n. d.	n. d.	3.8 ± 0.07	n. d.	n. d.	n. d.	n. d.	n. d.	3.0 ± 0.04	2.2 ± 0.04	2.7 ± 0.03	n. d.
Total AAs	28.7 ± 0.71	25.7 ± 0.07	22.2 ± 0.22	19.0 ± 0.04	33.8 ± 0.06	30.5 ± 0.04	37.6 ± 0.21	35.4 ± 0.05	44.2 ± 0.38	47.9 ± 0.04	51.7 ± 0.52	24.6 ± 0.07	38.8 ± 0.05	31.9 ± 0.11
Essential AAs	16.6 ± 0.41	14.6 ± 0.04	12.0 ± 0.08	10.8 ± 0.06	22.7 ± 0.05	18.7 ± 0.06	21.8 ± 0.04	22.5 ± 0.04	17.0 ± 0.15	18.3 ± 0.04	30.4 ± 0.23	16.5 ± 0.04	17.2 ± 0.05	21.7 ± 0.12

n. d.: not detected; Ala: alanine; Arg: arginine; Asp: aspartic acid; Glu: glutamic acid; Gly: glycine; His: histidine; Ile: isoleucine; Leu: leucine; Lys: lysine; Phe: phenylalanine; Ser: serine; Thr: threonine; Tyr: tyrosine; Val: valine; Total AAs: total amino acids; Essential AAs: essential amino acids; PPF80: pea protein flour (80% protein); PPF60: pea protein flour and wheat bran (60% protein); PPF40: pea protein flour and wheat bran (40% protein); PPF20: pea protein flour and wheat bran (20% protein); RPF80: rice protein flour (80% protein); RPF60: rice protein flour and wheat bran (60% protein); RPF40: rice protein flour and wheat bran (40% protein); RPF20: rice protein flour and wheat bran (20% protein); SLF40: sweet lupine flour and wheat bran (40% protein); SLF20: sweet lupine flour and wheat bran (20% protein); CF10: cassava flour and wheat bran (10% protein); PF10: potato flakes and wheat bran (10% protein); WB: wheat bran (control); Start: larvae at the beginning of the experiment.

## Data Availability

The data presented in this study are available on request from the corresponding author.

## Supplementary Materials

The following supporting information can be downloaded at: https://www.mdpi.com/article/10.3390/insects14030261/s1, Table S1: Nutritional composition (as specified by the manufacturer) of substrates on a fresh weight (FW) basis (%) used for *Tenebrio molitor* diets; Table S2: Amino acid composition of the substrates on a dry matter (DM) basis (%) used for *Tenebrio molitor* diets; Table S3: Orthogonal contrasts (*p*-values) of growth performance, feed and protein utilization efficiency and survival rate of *Tenebrio molitor* larvae of different feeding groups; Table S4: Orthogonal contrasts (*p*-values) of nutritional composition of *Tenebrio molitor* larvae.
