# Supplementary material for: Influence of Dietary Protein Content on the Nutritional Composition of Mealworm Larvae (Tenebrio molitor L.)
Source: Insects. 2023 Mar 6;14(3):261. doi: 10.3390/insects14030261 (PMC10053216; doi:10.3390/insects14030261)
Supplement: Supplementary file 1 [file insects-14-00261-s001.zip › insects-2221679-supplementary matterials.pdf]

insects

MDPI

Supplementary Material

Table S1. Nutritional composition (as specified by the manufacturer) of substrates on a fresh weight (FW) basis (%) used for *Tenebrio molitor* diets.

Substrate	Moisture (%)	Protein (% of FW)	Fat (% of FW)	Carbohydrate (% of FW)	Fiber (% of FW)	Ash (% of FW)	Manufacturer
Cassava flour	5.9	1.0	0.0	86.0	6.0	0.3	EWL Naturprodukte Handelsagentur AG, Randbach-Baumbach, Germany
Pea protein flour	2.7	80.0	8.0	4.9	4.2	0.2	Raab Vitalfood GmbH, Rohrbach, Germany
Potato flakes	9.2	8.3	0.5	76.2	4.6	1.2	Mühlenlädle, Kirchberg an der Murr, Germany
Rice protein flour	3.9	80.0	2.9	9.6	3.3	0.3	Raab Vitalfood GmbH, Rohrbach, Germany
Sweet lupine flour	5.6	43.0	12.0	10.0	28.0	1.4	Natura-Werk Gebr. Hiller GmbH & Co. KG, Hannover, Germany
Wheat bran	12.0	14.9	4.7	45.0	17.7	5.7	Roland Mills United GmbH & Co. KG, Bremen, Germany

Table S2. Amino acid composition of the substrates on a dry matter (DM) basis (%) used for *Tenebrio molitor* diets. Data are presented as mean ± standard deviation, $n = 2$.

Amino acid (% DM)	Pea protein flour (PPF)	Rice protein flour (RPF)	Sweet lupine flour (SLF)	Cassava flour (CF)	Potato flakes (PF)	Wheat bran (WB)
Ala	4.1 ± 0.0	4.0 ± 0.0	1.2 ± 0.0	0.1 ± 0.0	0.3 ± 0.0	0.6 ± 0.0
Arg	6.4 ± 0.0	8.2 ± 0.0	3.8 ± 0.0	0.1 ± 0.0	0.5 ± 0.0	0.8 ± 0.0
Asp	7.1 ± 0.0	11.3 ± 0.0	3.6 ± 0.0	0.1 ± 0.0	1.2 ± 0.0	1.0 ± 0.0
Glu	14.9 ± 0.0	18.2 ± 0.0	8.3 ± 0.0	0.2 ± 0.0	1.3 ± 0.0	3.5 ± 0.0
Gly	3.4 ± 0.0	3.7 ± 0.0	1.3 ± 0.0	0.1 ± 0.0	0.3 ± 0.0	0.6 ± 0.0
His	1.7 ± 0.0	2.1 ± 0.0	0.4 ± 0.0	0.1± 0.0	0.2 ± 0.0	0.3 ± 0.0
Ile	3.2 ± 0.0	4.5 ± 0.0	1.4 ± 0.0	0.1 ± 0.0	0.4 ± 0.0	0.4 ± 0.0
Leu	6.2 ± 0.0	8.0 ± 0.0	2.5 ± 0.0	0.1 ± 0.0	0.7 ± 0.0	1.0 ± 0.0
Lys	2.4 ± 0.0	6.3 ± 0.0	1.0 ± 0.0	0.1 ± 0.0	0.4 ± 0.0	0.3 ± 0.0
Phe	4.1 ± 0.0	5.3 ±0.0	1.2 ± 0.0	n. d.	0.4 ± 0.0	0.6 ± 0.0

Ser	3.9 ± 0.0	4.9 ± 0.0	1.9 ± 0.0	0.1± 0.0	0.3 ± 0.0	0.7 ± 0.0
Thr	2.7 ± 0.0	3.1 ± 0.0	1.2 ± 0.0	0.1± 0.0	0.4 ± 0.0	0.3 ± 0.0
Tyr	4.3 ± 0.0	4.0 ± 0.0	1.6 ± 0.0	0.1± 0.0	0.3 ± 0.0	0.4 ± 0.0
Val	4.6 ± 0.0	4.9 ± 0.0	1.3 ± 0.0	n. d.	0.4 ± 0.0	0.6 ± 0.0
Total AAs	69.0 ± 0.0	88.5 ± 0.0	30.7 ± 0.0	1.3 ± 0.0	7.1 ± 0.0	10.0 ± 0.0
Essential AAs	31.1 ± 0.0	42.4 ± 0.0	12.8 ± 0.0	0.6 ± 0.0	3.4 ± 0.0	4.3 ± 0.0

n. d.: not detected, Ala: alanine; Arg: arginine; Asp: aspartic acid; Glu: glutamic acid; Gly: glycine; His: histidine; Ile: isoleucine; Leu: leucine; Lys: lysine; Phe: phenylalanine; Ser: serine; Thr: threonine; Tyr: tyrosine; Val: valine; Total AAs: total amino acids.; Essential AAs: essential amino acids.

Table S3. Orthogonal contrasts (*p*-values) of larval weight gain per larvae (LWGpL), specific growth rate (SGR), food conversion ration (FCR), survival rate and protein efficiency ratio (PER) of *Tenebrio molitor* larvae of different feeding groups.

	LWGpL		SGR		FCR		Survival rate		PER	
**Groups**	*p*-value linear	*p*-value quadratic	*p*-value linear	*p*-value quadratic	*p*-value linear	*p*-value quadratic	*p*-value linear	*p*-value quadratic	*p*-value linear	*p*-value quadratic
WB vs. PPF80	<0.001	<0.001	<0.001	<0.001	<0.001	<0.001	>0.05	>0.05	<0.001	<0.001
WB vs. PPF60	<0.001	<0.001	<0.001	<0.001	>0.05	>0.05	>0.05	>0.05	<0.001	<0.001
WB vs. PPF40	<0.001	<0.001	<0.001	<0.001	>0.05	>0.05	>0.05	>0.05	<0.001	<0.001
WB vs. PPF20	<0.001	<0.001	<0.001	<0.001	>0.05	>0.05	>0.05	>0.05	<0.001	<0.001
WB vs. RPF80	<0.001	<0.001	<0.01	<0.01	>0.05	>0.05	<0.01	<0.01	<0.001	<0.001
WB vs. RPF60	<0.001	<0.001	<0.01	<0.01	>0.05	>0.05	>0.05	>0.05	<0.001	<0.001
WB vs. RPF40	<0.001	<0.001	>0.05	>0.05	>0.05	>0.05	>0.05	>0.05	<0.001	<0.001

WB vs. RPF20	<0.001	<0.001	>0.05	>0.05	<0.05	<0.05	>0.05	>0.05	<0.001	<0.001
WB vs. SLF40	<0.001	<0.001	>0.05	>0.05	<0.001	<0.001	>0.05	>0.05	<0.001	<0.001
WB vs. SLF20	<0.001	<0.001	>0.05	>0.05	>0.05	>0.05	>0.05	>0.05	<0.001	<0.001
WB vs. CF10	<0.001	<0.001	<0.01	<0.01	<0.01	<0.01	>0.05	>0.05	<0.001	<0.001
WB vs. PF10	<0.001	<0.001	>0.05	>0.05	>0.05	>0.05	>0.05	>0.05	<0.001	<0.001
PF10 vs. PPF80	<0.001	<0.001	<0.001	<0.001	<0.001	<0.001	>0.05	>0.05	<0.001	<0.001
PF10 vs. PPF60	<0.001	<0.001	<0.001	<0.001	>0.05	>0.05	>0.05	>0.05	<0.001	<0.001
PF10 vs. PPF40	<0.001	<0.001	<0.001	<0.001	>0.05	>0.05	>0.05	>0.05	<0.001	<0.001
PF10 vs. PPF20	<0.001	<0.001	<0.001	<0.001	>0.05	>0.05	>0.05	>0.05	<0.001	<0.001
PF10 vs. RPF80	<0.001	<0.001	<0.01	<0.01	>0.05	>0.05	<0.01	<0.01	<0.001	<0.001
PF10 vs. RPF60	<0.001	<0.001	<0.01	<0.01	>0.05	>0.05	>0.05	>0.05	<0.001	<0.001
PF10 vs. RPF40	<0.01	<0.05	>0.05	>0.05	>0.05	>0.05	>0.05	>0.05	<0.001	<0.001
PF10 vs. RPF20	>0.05	>0.05	>0.05	>0.05	>0.05	>0.05	>0.05	>0.05	>0.05	>0.05
PF10 vs. SLF40	<0.05	<0.05	>0.05	>0.05	>0.05	>0.05	>0.05	>0.05	<0.001	<0.001
PF10 vs. SLF20	>0.05	>0.05	>0.05	>0.05	>0.05	>0.05	>0.05	>0.05	>0.05	>0.05
PF10 vs. CF10	<0.01	<0.01	<0.01	<0.01	<0.001	<0.001	>0.05	>0.05	<0.001	<0.001
CF10 vs. PPF80	<0.001	<0.001	<0.001	<0.001	>0.05	>0.05	>0.05	>0.05	<0.001	<0.001
CF10 vs. PPF60	<0.01	<0.01	<0.05	<0.05	>0.05	>0.05	>0.05	>0.05	<0.001	<0.001

CF10 vs. PPF40	<0.01	<0.01	>0.05	>0.05	<0.01	<0.01	>0.05	>0.05	<0.001	<0.001
CF10 vs. PPF20	<0.01	<0.01	>0.05	>0.05	<0.01	<0.01	>0.05	>0.05	<0.001	<0.001
CF10 vs. RPF80	<0.05	<0.05	>0.05	>0.05	>0.05	>0.05	<0.01	<0.01	>0.05	>0.05
CF10 vs. RPF60	<0.01	<0.01	>0.05	>0.05	<0.01	<0.01	>0.05	>0.05	>0.05	>0.05
CF10 vs. RPF40	<0.01	<0.01	<0.01	<0.01	<0.01	<0.01	>0.05	>0.05	>0.05	>0.05
CF10 vs. RPF20	<0.01	<0.01	<0.01	<0.01	<0.01	<0.01	>0.05	>0.05	<0.001	<0.001
CF10 vs. SLF40	<0.01	<0.01	<0.01	<0.01	<0.001	<0.001	>0.05	>0.05	>0.05	>0.05
CF10 vs. SLF20	<0.01	<0.01	<0.01	<0.01	<0.001	<0.001	>0.05	>0.05	<0.001	<0.001
SLF20 vs. PPF80	<0.001	<0.001	<0.001	<0.001	<0.001	<0.001	>0.05	>0.05	<0.001	<0.001
SLF20 vs. PPF60	<0.001	<0.001	<0.001	<0.001	>0.05	>0.05	>0.05	>0.05	<0.001	<0.001
SLF20 vs. PPF40	<0.05	<0.05	<0.01	<0.01	>0.05	>0.05	>0.05	>0.05	<0.001	<0.001
SLF20 vs. PPF20	<0.001	<0.001	<0.01	<0.01	>0.05	>0.05	>0.05	>0.05	<0.001	<0.001
SLF20 vs. RPF80	<0.01	<0.01	<0.001	<0.001	>0.05	>0.05	<0.01	<0.01	<0.001	<0.001
SLF20 vs. RPF60	<0.01	<0.01	<0.01	<0.01	>0.05	>0.05	>0.05	>0.05	<0.001	<0.001
SLF20 vs. RPF40	<0.01	<0.01	>0.05	>0.05	>0.05	>0.05	>0.05	>0.05	<0.001	<0.001
SLF20 vs. RPF20	>0.05	>0.05	>0.05	>0.05	>0.05	>0.05	>0.05	>0.05	>0.05	>0.05
SLF20 vs. SLF40	<0.05	<0.05	>0.05	>0.05	>0.05	>0.05	>0.05	>0.05	<0.001	<0.001
SLF40 vs. PPF80	<0.001	<0.001	<0.001	<0.001	<0.001	<0.001	>0.05	>0.05	<0.001	<0.001
SLF40 vs. PPF60	<0.001	<0.001	<0.001	<0.001	<0.001	<0.001	>0.05	>0.05	<0.001	<0.001
SLF40 vs. PPF40	<0.001	<0.001	<0.001	<0.001	<0.01	<0.01	>0.05	>0.05	<0.01	<0.01

SLF40 vs. PPF20	<0.001	<0.001	<0.01	<0.01	>0.05	>0.05	>0.05	>0.05	>0.05	>0.05
SLF40 vs. RPF80	<0.001	<0.001	<0.01	<0.01	<0.01	<0.01	<0.01	<0.01	>0.05	>0.05
SLF40 vs. RPF60	<0.001	<0.001	<0.05	<0.05	<0.01	<0.01	>0.05	>0.05	>0.05	>0.05
SLF40 vs. RPF40	>0.05	>0.05	>0.05	>0.05	<0.01	<0.01	>0.05	>0.05	>0.05	>0.05
SLF40 vs. RPF20	<0.05	<0.05	>0.05	>0.05	>0.05	>0.05	>0.05	>0.05	<0.001	<0.001
RPF20 vs. PPF80	<0.001	<0.001	<0.001	<0.001	<0.01	<0.01	>0.05	>0.05	<0.001	<0.001
RPF20 vs. PPF60	<0.001	<0.001	<0.001	<0.001	<0.01	<0.01	>0.05	>0.05	<0.001	<0.001
RPF20 vs. PPF40	<0.001	<0.001	<0.001	<0.001	<0.01	<0.01	>0.05	>0.05	<0.001	<0.001
RPF20 vs. PPF20	<0.001	<0.001	<0.001	<0.001	>0.05	>0.05	>0.05	>0.05	<0.001	<0.001
RPF20 vs. RPF80	<0.001	<0.001	<0.001	<0.001	<0.01	<0.01	<0.01	<0.01	<0.001	<0.001
RPF20 vs. RPF60	<0.001	<0.001	<0.01	<0.01	<0.01	<0.01	>0.05	>0.05	<0.001	<0.001
RPF20 vs. RPF40	<0.001	<0.001	>0.05	>0.05	<0.05	<0.05	>0.05	>0.05	<0.001	<0.001
RPF40 vs. PPF80	<0.001	<0.001	<0.001	<0.001	<0.001	<0.001	>0.05	>0.05	<0.001	<0.001
RPF40 vs. PPF60	<0.001	<0.001	<0.01	<0.01	>0.05	>0.05	>0.05	>0.05	<0.001	<0.001
RPF40 vs. PPF40	<0.001	<0.001	<0.01	<0.01	>0.05	>0.05	>0.05	>0.05	<0.001	<0.001
RPF40 vs. PPF20	<0.001	<0.001	<0.05	<0.05	>0.05	>0.05	>0.05	>0.05	>0.05	>0.05
RPF40 vs. RPF80	<0.001	<0.001	<0.001	<0.001	>0.05	>0.05	<0.01	<0.01	>0.05	>0.05
RPF40 vs. RPF60	<0.001	<0.001	<0.01	<0.01	>0.05	>0.05	>0.05	>0.05	>0.05	>0.05
RPF60 vs. PPF80	<0.001	<0.001	<0.001	<0.001	<0.001	<0.001	>0.05	>0.05	<0.01	<0.01
RPF60 vs. PPF60	<0.001	<0.001	<0.01	<0.01	>0.05	>0.05	>0.05	>0.05	<0.01	<0.01
RPF60 vs. PPF40	<0.001	<0.001	<0.05	<0.05	>0.05	>0.05	>0.05	>0.05	<0.01	<0.01
RPF60 vs. PPF20	>0.05	>0.05	>0.05	>0.05	>0.05	>0.05	>0.05	>0.05	>0.05	>0.05
RPF60 vs. RPF80	<0.001	<0.001	<0.001	<0.001	>0.05	>0.05	>0.05	>0.05	>0.05	>0.05

RPF80 vs. PPF80	<0.001	<0.001	>0.05	>0.05	>0.05	>0.05	<0.01	<0.01	<0.001	<0.001
RPF80 vs. PPF60	<0.001	<0.001	>0.05	>0.05	>0.05	>0.05	<0.01	<0.01	<0.001	<0.001
RPF80 vs. PPF40	>0.05	>0.05	>0.05	>0.05	>0.05	>0.05	<0.01	<0.01	<0.001	<0.001
RPF80 vs. PPF20	<0.001	<0.001	<0.001	<0.001	>0.05	>0.05	<0.01	<0.01	>0.05	>0.05
PPF20 vs. PPF80	<0.001	<0.001	<0.001	<0.001	<0.01	<0.01	>0.05	>0.05	<0.001	<0.001
PPF20 vs. PPF60	<0.001	<0.001	<0.001	<0.001	>0.05	>0.05	>0.05	>0.05	<0.001	<0.001
PPF20 vs. PPF40	<0.001	<0.001	<0.001	<0.001	>0.05	>0.05	>0.05	>0.05	<0.001	<0.001
PPF40 vs. PPF80	<0.001	<0.001	<0.001	<0.001	<0.01	<0.01	>0.05	>0.05	>0.05	>0.05
PPF40 vs. PPF60	<0.001	<0.001	>0.05	>0.05	>0.05	>0.05	>0.05	>0.05	>0.05	>0.05
PPF60 vs. PPF80	<0.001	<0.001	<0.01	<0.01	>0.05	>0.05	>0.05	>0.05	>0.05	>0.05

Table S4. Orthogonal contrasts (*p*-values) of nutritional composition of *Tenebrio molitor* larvae.

**Groups**	Moisture		Crude protein		Crude fat	
	p-value linear	*p*-value quadratic	*p*-value linear	*p*-value quadratic	*p*-value linear	*p*-value quadratic
WB vs. PPF80	<0.001	<0.001	<0.001	<0.001	<0.001	<0.001
WB vs. PPF60	<0.001	<0.001	<0.001	<0.001	<0.001	<0.001
WB vs. PPF40	>0.05	>0.05	<0.001	<0.001	<0.001	<0.01
WB vs. PPF20	<0.001	<0.001	<0.001	<0.001	>0.05	>0.05
WB vs. RPF80	>0.05	>0.05	<0.001	<0.001	<0.001	<0.001
WB vs. RPF60	>0.05	>0.05	>0.05	>0.05	<0.01	<0.01
WB vs. RPF40	<0.001	<0.001	>0.05	>0.05	<0.01	<0.01
WB vs. RPF20	<0.001	<0.001	>0.05	>0.05	>0.05	>0.05
WB vs. SLF40	<0.001	<0.001	<0.001	<0.001	>0.05	>0.05
WB vs. SLF20	<0.001	<0.001	<0.001	<0.001	<0.001	<0.001
WB vs. CF10	<0.001	<0.001	<0.001	<0.001	<0.001	<0.001
WB vs. PF10	<0.001	<0.001	<0.001	<0.001	<0.01	<0.01

PF10 vs. PPF80	<0.001	<0.001	<0.001	<0.001	<0.001	<0.001
PF10 vs. PPF60	<0.001	<0.001	<0.001	<0.001	<0.001	<0.001
PF10 vs. PPF40	<0.001	<0.001	<0.001	<0.001	<0.001	<0.001
PF10 vs. PPF20	<0.001	<0.001	<0.001	<0.001	<0.001	<0.001
PF10 vs. RPF80	<0.001	<0.001	<0.001	<0.001	<0.001	<0.001
PF10 vs. RPF60	<0.001	<0.001	<0.001	<0.001	<0.001	<0.001
PF10 vs. RPF40	<0.001	<0.001	<0.001	<0.001	<0.001	<0.001
PF10 vs. RPF20	<0.001	<0.001	<0.001	<0.001	<0.01	<0.01
PF10 vs. SLF40	>0.05	>0.05	<0.001	<0.001	<0.01	<0.01
PF10 vs. SLF20	>0.05	>0.05	<0.001	<0.001	<0.01	<0.01
PF10 vs. CF10	>0.05	>0.05	>0.05	>0.05	>0.05	>0.05
CF10 vs. PPF80	<0.001	<0.001	<0.001	<0.001	<0.001	<0.001
CF10 vs. PPF60	<0.001	<0.001	<0.001	<0.001	<0.001	<0.001
CF10 vs. PPF40	<0.001	<0.001	<0.001	<0.001	<0.001	<0.001
CF10 vs. PPF20	<0.001	<0.001	<0.001	<0.001	<0.001	<0.001
CF10 vs. RPF80	<0.001	<0.001	<0.001	<0.001	<0.001	<0.001
CF10 vs. RPF60	<0.001	<0.001	<0.001	<0.001	<0.001	<0.001
CF10 vs. RPF40	<0.001	<0.001	<0.001	<0.001	<0.001	<0.001
CF10 vs. RPF20	<0.001	<0.001	<0.001	<0.001	<0.01	<0.01
CF10 vs. SLF40	>0.05	>0.05	>0.05	>0.05	>0.05	>0.05
CF10 vs. SLF20	>0.05	>0.05	>0.05	>0.05	>0.05	>0.05
SLF20 vs. PPF80	<0.001	<0.001	<0.001	<0.001	<0.001	<0.001
SLF20 vs. PPF60	<0.001	<0.001	<0.001	<0.001	<0.001	<0.001
SLF20 vs. PPF40	<0.001	<0.001	>0.05	>0.05	<0.001	<0.001
SLF20 vs. PPF20	<0.001	<0.001	>0.05	>0.05	<0.01	<0.01
SLF20 vs. RPF80	<0.001	<0.001	<0.001	<0.001	<0.001	<0.001
SLF20 vs. RPF60	<0.001	<0.001	<0.001	<0.001	<0.001	<0.001
SLF20 vs. RPF40	<0.001	<0.001	<0.001	<0.001	<0.001	<0.001
SLF20 vs. RPF20	<0.001	<0.001	<0.001	<0.001	<0.01	<0.01
SLF20 vs. SLF40	>0.05	>0.05	>0.05	>0.05	<0.01	<0.01
SLF40 vs. PPF80	<0.001	<0.001	<0.001	<0.001	<0.001	<0.001
SLF40 vs. PPF60	<0.001	<0.001	<0.001	<0.001	<0.01	<0.01
SLF40 vs. PPF40	<0.001	<0.001	>0.05	>0.05	<0.01	<0.01
SLF40 vs. PPF20	<0.001	<0.001	>0.05	>0.05	>0.05	>0.05

SLF40 vs. RPF80	<0.001	<0.001	<0.001	<0.001	<0.001	<0.001
SLF40 vs. RPF60	<0.001	<0.001	<0.001	<0.001	<0.01	<0.01
SLF40 vs. RPF40	<0.001	<0.001	<0.01	<0.01	<0.01	<0.01
SLF40 vs. RPF20	<0.001	<0.001	<0.01	<0.01	>0.05	>0.05
RPF20 vs. PPF80	<0.001	<0.001	<0.001	<0.001	<0.001	<0.001
RPF20 vs. PPF60	<0.001	<0.001	<0.001	<0.001	<0.01	<0.01
RPF20 vs. PPF40	<0.001	<0.001	<0.001	<0.001	<0.01	<0.01
RPF20 vs. PPF20	>0.05	>0.05	<0.001	<0.001	>0.05	>0.05
RPF20 vs. RPF80	<0.001	<0.001	<0.001	<0.001	<0.01	<0.01
RPF20 vs. RPF60	<0.001	<0.001	>0.05	>0.05	<0.01	<0.01
RPF20 vs. RPF40	>0.05	>0.05	>0.05	>0.05	<0.01	<0.01
RPF40 vs. PPF80	<0.001	<0.001	<0.001	<0.001	<0.001	<0.001
RPF40 vs. PPF60	<0.001	<0.001	<0.001	<0.001	<0.001	<0.001
RPF40 vs. PPF40	<0.001	<0.001	<0.001	<0.001	>0.05	>0.05
RPF40 vs. PPF20	>0.05	>0.05	<0.001	<0.001	<0.001	<0.001
RPF40 vs. RPF80	<0.001	<0.001	<0.001	<0.001	<0.01	<0.001
RPF40 vs. RPF60	<0.001	<0.001	>0.05	>0.05	>0.05	>0.05
RPF60 vs. PPF80	<0.001	<0.001	<0.001	<0.001	<0.001	<0.001
RPF60 vs. PPF60	<0.001	<0.001	<0.001	<0.001	<0.001	<0.001
RPF60 vs. PPF40	>0.05	>0.05	<0.001	<0.001	>0.05	>0.05
RPF60 vs. PPF20	<0.001	<0.001	<0.001	<0.001	<0.001	<0.001
RPF60 vs. RPF80	>0.05	>0.05	<0.001	<0.001	<0.01	<0.01
RPF80 vs. PPF80	<0.001	<0.001	<0.001	<0.001	>0.05	>0.05
RPF80 vs. PPF60	<0.001	<0.001	<0.001	<0.001	>0.05	>0.05
RPF80 vs. PPF40	>0.05	>0.05	<0.001	<0.001	<0.001	<0.001
RPF80 vs. PPF20	<0.001	<0.001	<0.001	<0.001	<0.001	<0.001
PPF20 vs. PPF80	<0.001	<0.001	<0.001	<0.001	<0.001	<0.001
PPF20 vs. PPF60	<0.001	<0.001	<0.001	<0.001	<0.001	<0.001
PPF20 vs. PPF40	<0.001	<0.001	>0.05	>0.05	<0.001	<0.001
PPF40 vs. PPF80	<0.001	<0.001	<0.001	<0.001	<0.01	<0.01
PPF40 vs. PPF60	<0.001	<0.001	<0.001	<0.001	<0.01	<0.01
PPF60 vs. PPF80	>0.05	>0.05	>0.05	>0.05	>0.05	>0.05
Start vs. PPF80	<0.001	<0.001	<0.001	<0.001	<0.001	<0.001
Start vs. PPF60	<0.001	<0.001	<0.001	<0.001	<0.001	<0.001

Start vs. PPF40	<0.001	<0.001	>0.05	>0.05	>0.05	>0.05
Start vs. PPF20	<0.001	<0.001	>0.05	>0.05	<0.001	<0.001
Start vs. RPF80	<0.001	<0.001	<0.001	<0.001	<0.001	<0.001
Start vs. RPF60	<0.001	<0.001	<0.001	<0.001	>0.05	>0.05
Start vs. RPF40	<0.001	<0.001	<0.001	<0.001	>0.05	>0.05
Start vs. RPF20	<0.001	<0.001	<0.001	<0.001	<0.001	<0.001
Start vs. SLF40	>0.05	>0.05	>0.05	>0.05	<0.001	<0.001
Start vs. SLF20	>0.05	>0.05	>0.05	>0.05	<0.001	<0.001
Start vs. CF10	>0.05	>0.05	<0.001	<0.001	<0.001	<0.001
Start vs PF10	>0.05	>0.05	<0.001	<0.001	<0.001	<0.001
Start vs WB	<0.001	<0.001	<0.001	<0.001	<0.001	<0.001
